# The Role of Cardiovascular Surgery in the Management of a Patient Diagnosed With Congenital Cutis Laxa Syndrome Complicated by Multivalvular Heart Disease

**DOI:** 10.7759/cureus.19359

**Published:** 2021-11-08

**Authors:** Abdulrahman Saleh Aldairi, Mohammad Shihata, Abdulbadee A Bogis, Mohammad Alrefai, Uthman Aluthman, Ahmed Jamjoom

**Affiliations:** 1 Department of Surgery, Faculty of Medicine, Umm Al-Qura University, Makkah, SAU; 2 Division of Cardiac Surgery, Cardiovascular Department, King Faisal Specialist Hospital and Research Center, Jeddah, SAU

**Keywords:** cutis laxa, mitral valve regurgitation, tricuspid valve regurgitation, congenital cutis laxa, cardiopulmonary complications, tricuspid valve prolapse, mitral valve prolapse

## Abstract

Cutis laxa syndrome is an uncommon connective tissue disorder affecting the major ultrastructure of the skin by progressive loss of elasticity. The results of this syndrome lead to the appearance of premature aging, which might also affect the internal organs. The disorder can be either congenital or acquired. The congenital form consists of autosomal dominant, autosomal recessive, and X-linked recessive patterns. The autosomal recessive pattern is the most common and severe one. Different systemic complications have been described in congenital cutis laxa syndrome, but the most serious and lethal one is cardiopulmonary abnormalities. In this report, we discuss the presentation of congenital cutis laxa syndrome with successful cardiovascular surgical management of multiple valvular heart diseases.

## Introduction

Cutis laxa (CL) syndrome is defined as a rare connective tissue disorder with major characteristic features of wrinkled, redundant, sagging, and inelastic skin due to a major defect in elastin synthesis or abnormalities in the structure of the extracellular matrix [1-3]. It can be either congenital or acquired [4]. The congenital form is more common than the latter [5]. The major patterns of inheritance for this syndrome are autosomal dominant (ADCL), autosomal recessive (ARCL), and X-linked recessive (XLCL) [1,6-8]. The most common inherited pattern is ARCL, with severe complications involving the cardiovascular system, pulmonary system, musculoskeletal system, and intellectual functioning [4,9]. On the other hand, the ADCL pattern is multifarious in presentation between the onset of childhood and adult life [4]. Herein, we present a rare case of congenital CL (CCL) syndrome with severe mitral and tricuspid valve prolapse causing severe regurgitation of both valves and pulmonary hypertension in a 15-year-old female. We also discuss the best treatment strategy based on the available literature and our experience.

## Case presentation

**Figure 1:**
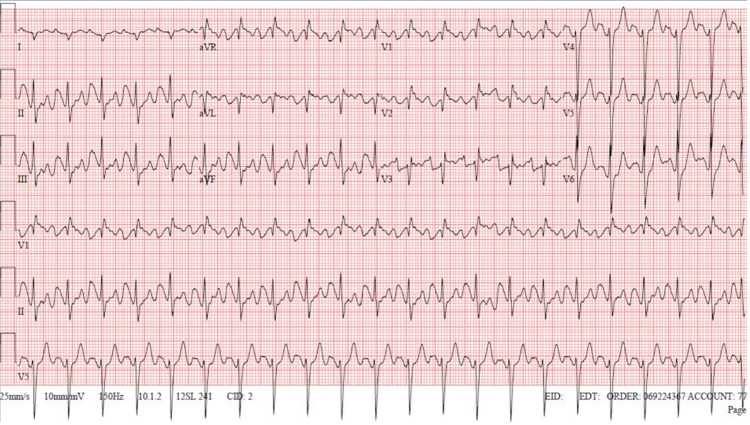
ECG revealed sinus tachycardia, right atrial enlargement, right ventricular hypertrophy, and prolonged QT interval.

**Figure 2:**
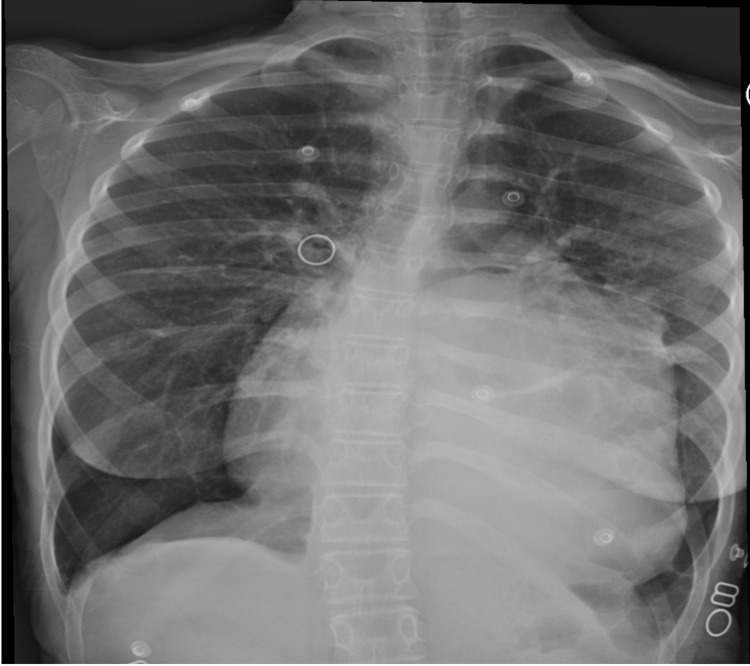
Chest x-ray revealed cardiac silhouette enlarged with hyperinflated lung and left retrocardiac airspace opacity with subsegmental atelectasis, as well as blunted bilateral costophrenic angles.

**Figure 3:**
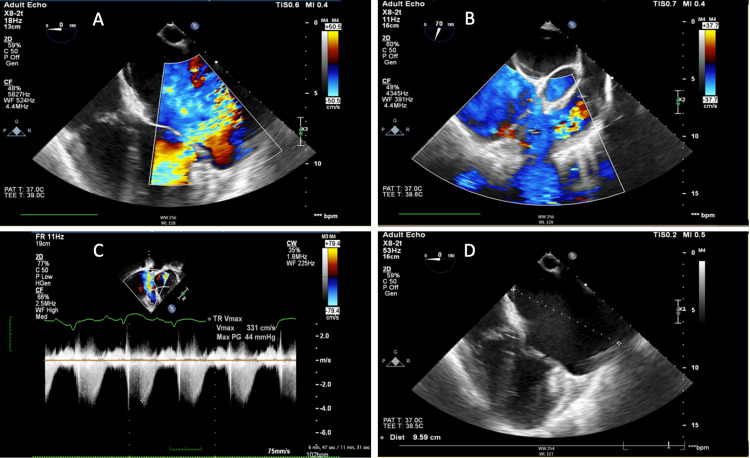
Preoperative TEE demonstrated (A) mitral valve regurgitation, (B) tricuspid valve regurgitation, (C) tricuspid valve regurgitation pressure gradient, and (D) enlargement of both atria with the measurement of the left atrium (9.59 cm).

A 15-year-old female patient was referred by a pediatric cardiologist to our pediatric cardiac surgery clinic with a confirmed diagnosis of CCL syndrome since birth by a dermatologist. Her cardiovascular symptoms started one month before the presentation with a history of recurrent episodes of shortness of breath, palpitations, and chest pain. The severity of the symptoms has increased in the past few weeks. At the time of referral, she was on furosemide 10 mg twice daily and enalapril 10 mg once daily. Her parents are phenotypically normal. All her siblings, five brothers and two sisters, are free from the disorder. Also, the patient has a remarkable family history, as her cousin is a 20-year-old male with the same disorder. There is consanguinity between parents in the family. On general examination, she had a senile appearance with generalized inelastic, loose, and sagging skin. Vital signs revealed a heart rate of 114 beats per minute, respiratory rate of 20 breaths per minute, blood pressure of 123/73 mmHg, oxygen saturation (SpO2) of 100% in room air, and temperature of 36 °C. On cardiac examination, the precordium was hyperactive, the first and second heart sounds were obscured, and pansystolic murmur grade III/VI radiating to the axilla was detected. The hematological studies were within normal limits. Electrocardiogram (ECG) showed sinus tachycardia with right atrial enlargement and right ventricular hypertrophy (Figure 1). Chest x-ray showed cardiomegaly with subsegmental atelectasis (Figure 2). For more assessment and operative plan, transesophageal echocardiogram (TEE) revealed severe mitral and tricuspid valve prolapse with malcoaptation causing severe regurgitation of both valves with pulmonary hypertension and severe dilatation of both right and left atria (Figures 3A-3D). After the patient’s condition was discussed in the heart team meeting, the plan was set for mitral and tricuspid valve repair versus replacement, depending on the intraoperative findings. Also, the patient was planned to be counseled by a medical geneticist. The case was discussed with the patient and her family as they were involved in the clinical decision. 

Procedure

**Figure 4:**
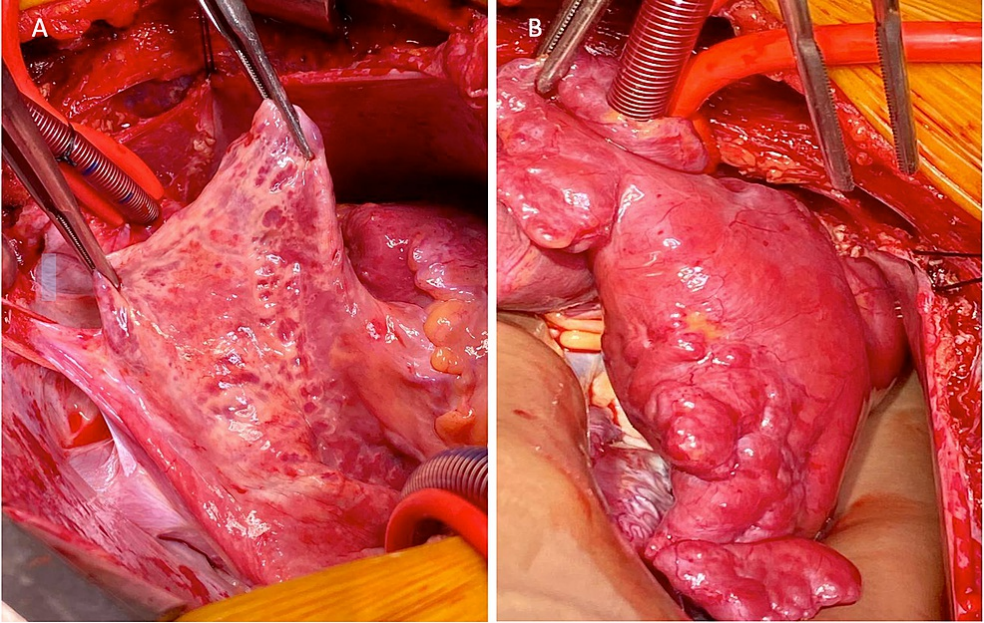
Intraoperative findings demonstrated (A) enlarged dilated right atrial appendage and (B) enlarged dilated left atrial appendage.

**Figure 5:**
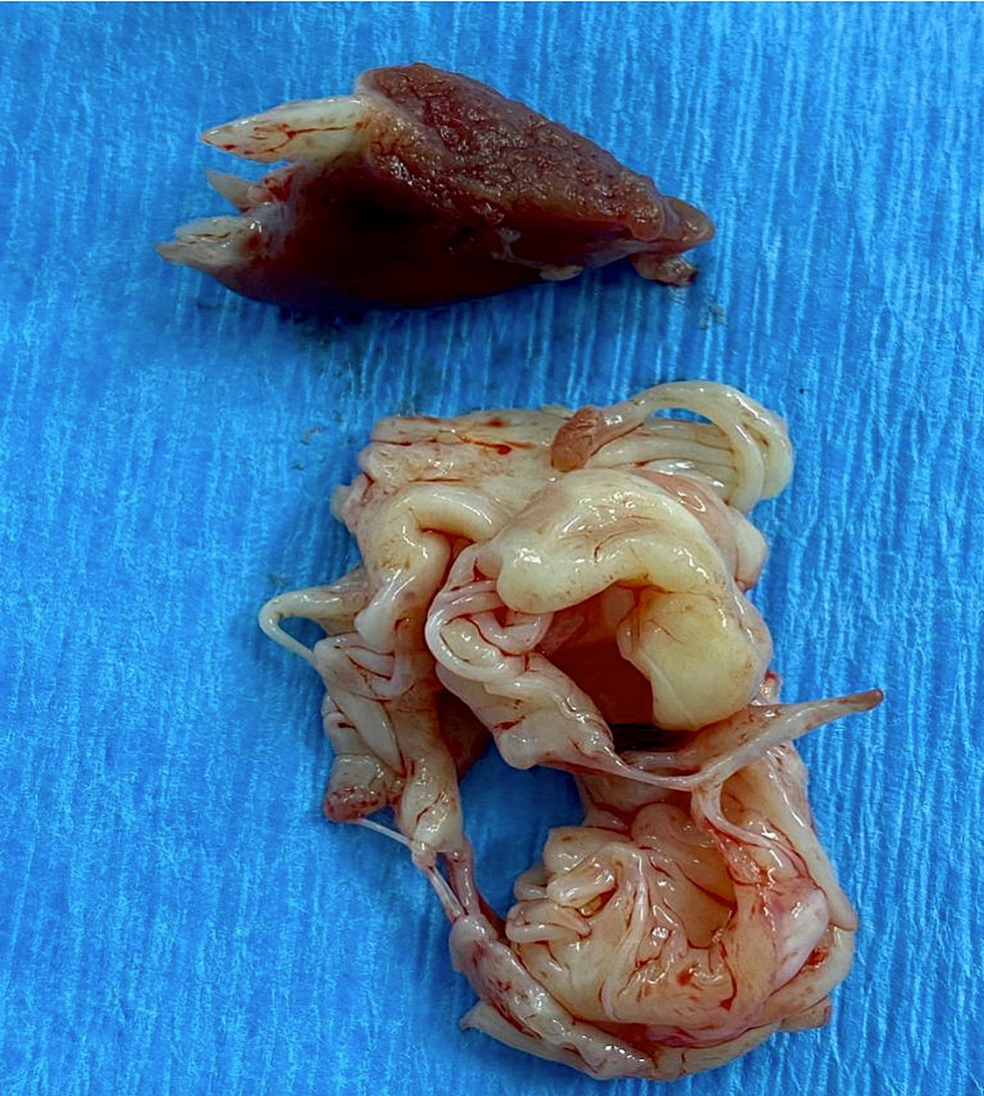
Elongated enlarged left papillary muscle and mitral valve leaflets.

Under general anesthesia, midline sternotomy was carried out, and the thymus was resected due to its enormous size. Standard cannulation was accomplished through the ascending aorta and superior and inferior vena cava with snugging around each cannula. Consequently, standard cardioplegic arrest with full flow of cardiopulmonary bypass (CPB) was achieved as the patient was cooled down to a temperature of 30 °C. The aortic valve was immediately examined after the aorta was transversely opened, and it had some significant enlargement and dilatation of the leaflet, but the valve was manually competent; thence, no intervention was done to the aortic valve. Both atria were significantly enlarged; thereupon, right and left atrial appendages were resected in combination with atrioplasty (Figures 4A, 4B). The left atrium was opened, the mitral valve was examined and showed significant myxomatous changes of both anterior and posterior leaflets, and the valve was irreparable. Hence, the decision was made intraoperatively to replace the mitral valve with a 33-mm St. Jude Medical Epic porcine valve prosthesis (Figure 5). After this, through an incision into the right atrium, the tricuspid valve was found to have a cleft at the septal leaflet and the anterior leaflet with significant dilation of the annulus. Accordingly, commissural tricuspid annuloplasty was performed. The tricuspid valve test rendered a competent valve. After completing the procedure, the patient was fully rewarmed and weaned off CPB. Intraoperative TEE showed trace tricuspid regurgitation, and the prosthetic valve was well seated at the mitral position. With these findings, the patient was decannulated, chest tubes and pacer wire were inserted, and closure was performed. She was transferred to the cardiac surgery intensive care unit (CSICU) in a stable condition. During the first 15 hours in the CSICU, the chest tubes drained blood in a total of 1,450 mL (right lower pleura), 300 mL (mediastinum), and 1,330 mL (left lower pleura). Afterward, the patient received five units of packed red blood cells (PRBCs), seven units of fresh frozen plasma (FFP), two units of cryoprecipitated antihemophilic factor (Cryo), two units of platelets, and two doses of intravenous protamine sulfate (50 mg per dose). The bleeding was then controlled (Table 1). The patient was shifted from the CSICU to the ward on postoperative day 3 (POD 3). All chest drains were removed subsequently, and laboratory work was within normal limits. Predischarge transthoracic echocardiogram (TTE) showed no significant changes, and the patient was planned to be discharged home on POD 6 in satisfactory condition.

 

**Table 1:** The amount of chest tubes drainage. *POD zero is defined as the same day of surgery. The sites of chest tubes: a: Right lower pleura. b: Mediastinum. c: Left lower pleura.

Postoperative Day (POD)	POD Zero*	POD One	
	15 hours	12 hours	24 hours
Chest tube 1a	1450 ml	110 ml	500 ml
Chest tube 2b	300 ml	80 ml	160 ml
Chest tube 3c	1330 ml	50 ml	220 ml

## Discussion

CCL syndrome is considered to be an extremely rare variant group of disorders [1]. CCL has different inheritance patterns [1]. Ordinarily, ADCL has a generally benign course with changes often limited to the skin [1]. These skin changes may appear at any age, and patients presenting in adulthood with ADCL have a normal life expectancy with usually no internal organ defects [10,11]. In contrast, the presentation of ADCL in infancy has different dimensions, including intrauterine growth retardation, delayed fontanelle closure, ligamentous laxity, and the loss of lung tissue elasticity resulting in pulmonary emphysema [12,13]. A range of various changes that might take place between the first two months of life and the end of the second year is composed of skin changes possibly anticipated by multiple episodes of edema and ending with a senile appearance [13]. CCL is inherited more frequently in the severe ARCL pattern [14]. ARCL has characteristic features, such as facies with downward slanting palpebral fissures, a wide flat nose, sagging cheeks, and large ears [11]. Moreover, the knees, abdomen, and thigh have prominent skin folds [11]. The prognosis and life expectancy in ARCL are determined by systemic organ involvement; commonly, the major portion of complications originates from cardiopulmonary abnormalities, such as pulmonary emphysema, cor pulmonale, right-sided heart failure, aortic aneurysm, and pulmonary artery stenosis [14]. Other complications include dislocation of the hips, joint hyperextensibility, hernia, hollow viscus diverticula, and osteoporosis [1]. Out of these complications of ARCL, pulmonary emphysema and cor pulmonale are the most common that may occur in the first month of life, and most patients die by the third year. Generally, the life span of these children with ARCL is short [1]. The inheritance pattern of XLCL, also known as occipital horn syndrome, is caused by a mutation in the ATP7A gene leading to dysfunction of the ATP7A protein, which is responsible for copper-transporting ATPase in all tissues except the liver. Also, this genetic mutation can result in a severe type of occipital horn syndrome called Menkes disease [2]. To our knowledge, two cases have been reported in the literature with CL syndrome that resulted in severe valvular heart disease plus other cardiovascular abnormalities and are considered to be candidates for surgical intervention with their available data being summarized in Table 2. The first case was reported by Roussin et al. of a 27-year-old male patient diagnosed with CL syndrome who had a resection of the right coronary sinus aneurysm, coronary reimplantation, and aortic valve replacement using 29-mm Freestyle (Medtronic) stentless porcine valve prosthesis [14]. The second one was reported by Szabo et al. where a 38-year-old male patient was diagnosed with CCL syndrome complicated by aneurysm of the sinuses of Valsalva and of ascending aorta requiring surgical repair; one year later, the patient developed severe aortic insufficiency and required aortic valve replacement [15]. Our case illustrates the severity of cardiovascular complications with mitral and tricuspid valve prolapse leading to severe regurgitation of both valves and pulmonary hypertension. Intraoperative findings were the most important factor in choosing between valve replacement or repair. In addition, our justification for using a porcine valve prosthesis instead of a mechanical valve in this patient is the social status, as this patient lives in a remote suburb area with difficult access to tertiary care centers making it difficult to comply with anticoagulant therapy. Furthermore, based on the literature, Karacan et al. demonstrated CCL syndrome to be a notoriously unexpectable disorder with multiple cases reported to have abrupt bleeding diathesis and defective wound healing [16]. To the best of our knowledge, this is the first case reported in the literature with successful surgical treatment of both mitral and tricuspid valves associated with this syndrome.

**Table 2:** A summary of cardiovascular surgical patients diagnosed with cutis laxa syndrome who had cardiovascular abnormalities in particular valvular heart disease. Abbreviations: CVS: cardiovascular system, y-o: year-old

Study	Age	CVS abnormalities	Procedure	Survival Outcome	Comments
Roussin [14]	27 y-o	1-Aneurysm of the right coronary sinus of Valsalva.	1-Resection of the right coronary sinus aneurysm.	Alive	-
		2-Occlusion of proximal right coronary artery.	2-Coronary reimplantation.		
		3-Severe aortic regurgitation.	3-Aortic valve replacement.		
Szabo [15]	38 y-o	1-Aneurysm of the sinuses of Valsalva.	1-Valvoplasty.	-	-
		2-Aneurysm of the ascending aorta.	2-Aortic graft.		
		3-Severe aortic regurgitation.	3-Aortic valve replacement.		
Our patient	15 y-o	1-Enlarged thymus gland.	1-Thymus resection.	Alive	Postoperative bleeding from chest tubes
		2-Mitral valve prolapse with regurgitation.	2-Mitral valve replacement.		
		3-Tricuspid valve prolapse with regurgitation.	3-Tricuspid valve repair.		
		4-Enlargement of right and left atrial appendages.	4-Resection of both atrial appendages.		
		5-Enlargmnet of both atria.	5-Atrioplasty.		
		6-Pulmonary hypertension.			

## Conclusions

From a cardiovascular surgical point of view, awareness of the systemic complications in CL syndrome is important, particularly cardiopulmonary complications. Early referral of these patients to receive medical and surgical treatment in a specialized medical center would be the main course to determine the prognosis and to avoid more systemic complications. Further research projects are required to determine the association between the different genetic subgroups in cardiac valvular disease and the long-term outcomes.
